# The Optimized Fabrication of a Novel Nanobubble for Tumor Imaging

**DOI:** 10.3389/fphar.2019.00610

**Published:** 2019-05-31

**Authors:** Jiaqi Zhang, Yihan Chen, Cheng Deng, Li Zhang, Zhenxing Sun, Jing Wang, Yali Yang, Qing Lv, Wei Han, Mingxing Xie

**Affiliations:** ^1^Department of Ultrasound, Union Hospital, Tongji Medical College, Huazhong University of Science and Technology, Wuhan, China; ^2^Hubei Province Key Lab of Molecular Imaging, Wuhan, China

**Keywords:** nanobubbles, ultrasound, contrast imaging, tumor, fabrication

## Abstract

Nanobubbles with a size of less than 1 µm can be used as ultrasound contrast agents for diagnosis and as drug/gene carriers for therapy. However, the optimal method of preparing uniform-sized nanobubbles is considered controversial. In this study, we developed novel biocompatible nanobubbles by performing differential centrifugation to isolate the relevant subpopulation from the parent suspensions. Compared with the method of modulating the thickness of the phospholipid film without centrifugation, nanobubbles fabricated under optimal centrifugation conditions exhibited a uniform bubble size, good stability, and low toxicity. Using *in vitro* ultrasound imaging, nanobubbles displayed excellent enhancement ability, which was comparable to microbubbles. In an *in vivo* experiment, the video intensity of nanobubbles in tumors was stronger than that of microbubbles at different times (5 min, 163.5 ± 8.3 a.u. vs. 143.2 ± 7.5 a.u., *P* < 0.01; 15 min, 125.4 ± 5.2 a.u. vs. 97.3 ± 4.6 a.u., *P* < 0.01). Fluorescence imaging obtained by confocal laser scanning microscopy demonstrated that obviously more nanobubbles passed through the vessel wall into the extravascular and intercellular space of tumors, compared with microbubbles. In conclusion, this optimized preparation method will strongly promote the application of nanobubbles in imaging and therapy.

## Introduction

Molecular imaging has undergone explosive growth since it emerged in the early 21st century. This technique is used not only to visualize the cellular functions in tissues and organs but also to monitor molecular processes in living organisms without disturbing these processes (Weissleder, 2006; Hussain and Nguyen, 2014; Keliher et al., 2017). As an important branch of molecular imaging, ultrasound-based molecular imaging has been extensively used in both experimental studies and clinical practices (Willmann et al., 2008b; Liu et al., 2015; Willmann et al., 2017).

Currently, the most used ultrasound contrast agents (UCAs) are microbubbles, with diameters ranging from 1 to 10 µm (Zhang et al., 2017). Microbubbles are widely used in molecular imaging of angiogenesis (Willmann et al., 2008a; Wu et al., 2011), inflammation (Machtaler et al., 2015; Liao et al., 2017), thrombi (Lu et al., 2016; Zhu et al., 2016), plaques (Zhang et al., 2016), and so on (Frauscher et al., 2001). But limited by the particle size, microbubbles cannot pass through the vessel wall and just served as blood pool agents (Ferrara et al., 2009; Moestue et al., 2012). To address this challenge, nanobubbles have attracted considerable attention.

**Figure 1:**
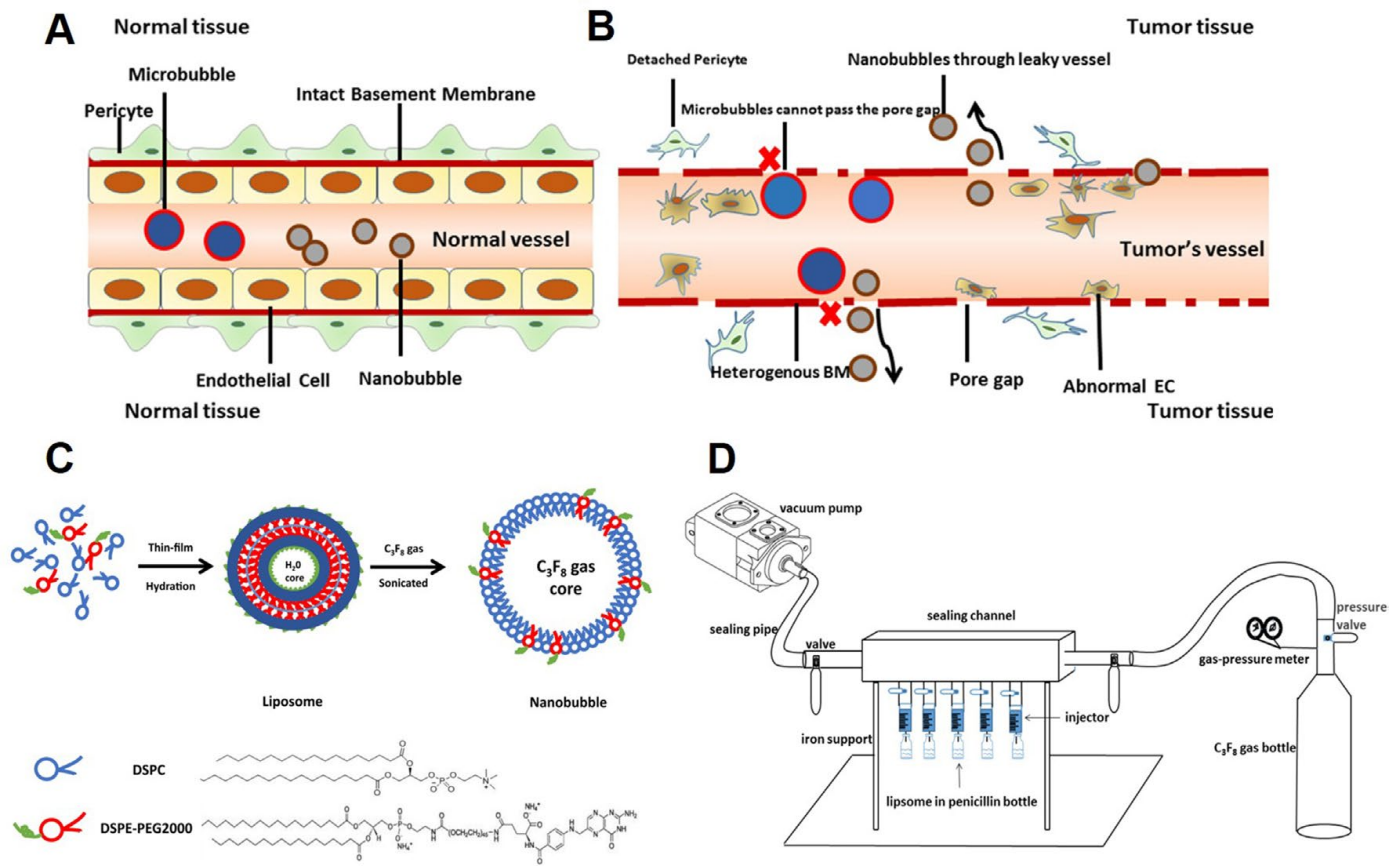
Schematic illustration of this study. **(A)** Neither microbubbles nor nanobubbles can pass through the endothelial gap in normal tissues. **(B)** Microbubbles cannot pass the endothelial gap but nanobubbles can in tumor tissues. **(C)** The procedure for fabrication of nanobubbles. **(D)** The homemade setup for exchanging octafluoropropane (C_3_F_8_) gas.

Due to their nanoscale size, nanobubbles have great potential application in extravascular molecular imaging, especially in tumors (Maeda et al., 2009; Rapoport et al., 2009). In normal tissues, the vascular endothelial gap is less than 7 nm (Hobbs et al., 1998). Thus, the vast majority of particles cannot pass freely (**Figure 1A**). However, in tumors, the vascular endothelial gap is approximately 380–780 nm (Maeda et al., 2009). Nanobubbles could permeate through the vasculature and get into extravascular and intercellular space (**Figure 1B**). Furthermore, because of the EPR (enhanced permeability and retention) effect, nanobubbles exhibit exaggerated extravasation and retention in tumors. Therefore, nanobubbles have been applied in tumor-targeted imaging and therapy widely (Rosen et al., 2012; Gao et al., 2017; Song et al., 2017).

Many studies have reported the fabrication of nanobubbles (Zong et al., 2006; Krupka et al., 2010; Yin et al., 2012). Among these, nanobubbles composed of a phospholipid shell and a gas core are considered to have optimal contrast enhancement ability (**Figure 1C**). However, the preparation of uniform-sized nanobubbles has been controversial. Some studies showed that the centrifugal condition was the key factor that affects the diameter of nanobubbles (Yin et al., 2012), but others reported that phospholipid film thickness was critical in the determination of the diameter of nanobubbles (Liao et al., 2014; Cai et al., 2015). Until now, few studies have carefully analyzed the two types of methods.

In this study, we focused on the impact of centrifugal condition on the preparation of nanobubbles. The physical characteristics of nanobubbles produced by optimal centrifugal condition were investigated. At the same time, the morphology, particle size, and polydispersity index (PDI) of nanobubbles prepared by centrifugation and controlling phospholipid film thickness were compared.

## Materials and Methods

### Materials

Phospholipids such as 1,2-distearoyl-sn-glycerol-3-phosphatidylcholine (DSPC) and 1,2-distearoyl-sn-glycerol-3-phosphoethanolamine *N*-[biotinyl(polyethyleneglycol) 2000] (DSPE–PEG 2000) were purchased in powder form (Avanti Polar Lipids Inc., Alabaster, AL). Octafluoropropane (C_3_F_8_) gas was purchased from the R&D Center for Specialty Gases at the Research Institute of Physical and Chemical Engineering of Nuclear Industry (Tianjin, China). The fluorescent dye DiI was purchased from Beyotime (Haimen, China). The Cell Counting Kit-8 (CCK-8) was purchased from Dojindo (Japan). BALB/c mice (8–10 weeks old) and Sprague–Dawley (SD) rats (weight, 200–220 g) were purchased from the Animal Breeding and Research Center of Tongji Medical College, Huazhong University of Science and Technology, China. All animals were treated according to the policy and regulations approved by the Huazhong University of Science and Technology Animal Care and Use Committee.

### Synthesis of Bubbles

We used two different methods to prepare nanobubbles. First, nanobubbles were prepared by controlling phospholipid film thickness, according to the previous study (Cai et al., 2015). In brief, fixed-ratio (molar ratios = 9:1) mixtures of DSPC and DSPE-PEG 2000 (5 mg, 10 mg, 15 mg, 20 mg, or 25 mg) were dissolved in chloroform. A small amount of red fluorescent membrane probe DiI was added. Then, the solvent was removed under nitrogen flow at room temperature, followed by vacuum treatment over 2 h. The dry lipid films were hydrated with a buffer solution consisting of 80% Tris (0.1 M, pH 7.4), 10% glycerol, and 10% propylene glycol (v/v) in a tube. Then, the tube was placed in a water bath at 55–60°C and treated by ultrasonic cleaner at 40 kHz for 10–15 min, until the films completely dissolved. The resulting solution was subpackaged into 4-mL vials (1 mL each vial) sealed by rubber caps. Finally, the air in the vial was exchanged with C_3_F_8_ using a homemade equipment (**Figure 1D**). Bubbles were formed by shaking the vial with a vibrator for 30 s.

Second, nanobubbles were prepared by centrifugation. A total of 15 mg of DSPC: DSPE-PEG 2000 in the molar ratio 9:1 was dissolved in chloroform. The following steps included thin-film formation, hydration, and sonication, just the same as the previous steps. After bubble mixtures were formed by the vibrator, different centrifugation speeds (20 g, 70 g, 140 g, and 400 g) were subsequently applied for 3 min. Small nanobubbles were collected after collecting the lower liquid layer. Finally, the nanobubbles were resuspended and stored at 4°C.

The concentration of nanobubbles was calculated using a hemacytometer. All measurements were carried out in triplicate and averaged.

### Particle Sizing and Zeta Potential Measurements

The particle sizes were measured by dynamic light scattering (DLS) using a Delsa^™^ Nano (Beckman Instruments Corporation). In total, 10 µL of the sample and 90 µL of phosphate buffer saline (PBS) were mixed in sample wells before measuring the particle sizes at 25°C. The zeta potential of each sample was measured using a Zeta Analyzer (Beckman Instruments Corporation) to determine the electrophoretic light scattering at 25°C. All samples used for the zeta potential measurements were prepared at the same concentration as those used for particle sizing. The particle size of each sample was measured three times.

### Morphology and Stability of Nanobubbles

The nanobubbles solution was diluted threefold and well mixed. Subsequently, a 5-mL suspension was dropped onto a transmission electron microscope (TEM) grid, negatively stained with 2% phosphotungstic acid, and allowed to rest for 6 h at room temperature. The morphologies and structures of the nanobubbles were then observed by TEM (Hitachi H-7500, Hitachi Limited, Tokyo, Japan). The particle size of the nanobubbles was calculated at 1, 3, 7, 10, and 14 days, and the concentration of the nanobubbles was examined after storage at 4°C for 1, 2, 3, 4, 5, and 6 h.

### *In Vitro* Biocompatibility Tests and Cytotoxicity Assay

We have conducted an experimental study on the biocompatibility of nanobubbles (NBs). Briefly, bEnd3 (mouse brain endothelial) cells were chosen to evaluate the cytotoxicity of the NBs. The cells were seeded at a density of 5,000 cells/well in 96-well plates and then cultured in 100 µL of Roswell Park Memorial Institute-1640 (RPMI-1640) medium containing 10% fetal bovine serum (FBS, Biological Industries, Israel) in an incubator with 5% CO_2_ for 24 h. The cells were subsequently incubated with gradient concentrations of nanobubbles (from 1 × 10^5^ to 1 × 10^9^ bubbles/mL) for 8 h. The medium was then replaced with 100 µL of fresh medium containing 10 µL of CCK-8 solution (Dojindo, Japan). Afterward, the cells were incubated for another 4 h. The absorbance of each well at 450 nm was recorded using an Infinite F200 multimode plate reader (Tecan, Männedorf, Switzerland). All experiments were carried out in triplicate.

### *In Vivo* Safety and Toxicity Evaluations of Nanobubbles

To evaluate the potential toxicity and adverse effects of the nanobubbles, all rats were continuously observed for relevant indices such as appearance, independent activity, and mortality. Animal experiments were approved by the Animal Care and Use Committee of Huazhong University of Science and Technology. After the rats were sacrificed, the main organs (i.e., the heart, lung, liver, spleen, and kidney) were harvested and fixed in 4% paraformaldehyde. These tissues were sectioned and stained with hematoxylin and eosin (HE) for histopathological examination. A total of 25 healthy SD rats were randomly divided into five groups (*n* = 5 per group). The control group did not receive any injection, and the other four groups were injected intravenously with PBS, DSPC (dose, 1.2 mg/kg), DSPE-PEG 2000 (dose, 1.2 mg/kg), or NBs (10^9^ NBs). These animals were sacrificed 24 h after the injections. Their heart, liver, spleen, lungs, and kidneys were then fixed, embedded in paraffin, and cut into 5-µm-thick sections for HE staining. Images were collected using an Olympus light microscope.

### *In Vitro* Ultrasound Imaging

To compare the ultrasonic imaging ability of the nanobubbles and microbubbles, *in vitro* ultrasound imaging experiments were performed. Briefly, 1 mL of NB or MB suspension at various bubble concentrations (from 1.0 × 10^5^ to 1.0 × 10^9^ bubbles/mL) was added to the sample wells of a homemade 2% (w/v) agarose mold. A clinical ultrasound scanner (Philips IU Elite) system with an L12-5 high-frequency linear transducer was used. Mechanical index (MI) was 0.10. The focal zone was placed at a depth of 1.5 cm, which was at the center of the sample well. Three images of each sample were taken. ImageJ software was used to analyze the grayscale values of the samples. The quantitative grayscale ultrasonic intensity of the samples was normalized to that of gas-free water. The intensity value was defined as the ratio of the grayscale value of the contrast agent to that of gas-free water.

### *In Vivo* Contrast-Enhanced Ultrasound Imaging in Rats

The *in vivo* imaging capability of the nanobubble contrast agents was evaluated using SD rats. Each rat was anesthetized with 300 mg/kg of 10% chloral hydrate by intraperitoneal injection. The animals were placed on a warm blanket to maintain their body temperatures. In order to compare the performance of nanobubbles with microbubbles, 150 μL of nanobubble suspension (10^9^ bubbles/mL) or microbubbles at the same amount were intravenously injected into the same mice in random order. A 1-h waiting time was allowed to clear contrast agents from previous injections. The left ventricular opacification (LVO) was conducted using a broadband L12-5 high-frequency linear transducer in contrast mode with an MI of 0.07.

### *In Vivo* Passive Tumor-Targeting Ultrasound Imaging in Mice

CT26 cells were transplanted into BALB/c mice as a xenograft model. The cells had been previously cultured in RPMI-1640 medium supplemented with 10% FBS (GIBCO, Carlsbad, CA) at 37°C and 5% CO_2_. A total of 10 BALB/c xenograft model mice (4–5 weeks old, 18–20 g) were examined. The cells (10^7^) were suspended in phosphate-buffered saline and subcutaneously injected into the right flanks (at the level of the liver) for tumor xenografts. All *in vivo* experiments began when the tumors reached a diameter of 0.8–1.0 cm. Mice were anesthetized with 10% chloral hydrate and fixed on a plate before ultrasonic imaging. To decrease speckle variance, both the ultrasound probe and the animal were fixed and remained at the same position throughout the study. As described above, nanobubbles and microbubbles were used in the same mice to compare the performance. The interval between two injections was 2 h. Ultrasonic images were acquired by a PHILIPS IU22 ultrasound system with a 9–12 MHz linear probe. All digital clips and images were stored for offline examination. Grayscale images were analyzed using ImageJ (v1.37; National Institutes of Health, Bethesda, MA).

### Confocal Laser Scanning Microscopy Examination

In order to confirm that nanobubbles were small enough to pass through the endothelial gaps in tumors, we used confocal laser scanning microscopy (CLSM) to determine the location of red fluorescently dyed nanobubbles *in vivo*. Tumor-bearing mice were randomly separated into two groups. One group was injected with DiI-labeled nanobubbles, and the other was injected with DiI-labeled microbubbles. After bubble injection, the heart of each mouse was perfused with 0.9% normal saline until the labeled bubbles were cleared from circulation. The tumors and muscles of the right thigh (used as negative controls because the endothelial cell connections are continuous in skeletal muscles) were immediately extracted and sectioned into 5-μm slices. To visualize the vessels in tumors, slices were incubated with rat anti-mouse CD31 antibody (eBioscience, San Diego, CA) at a dilution of 1:200 overnight at 4°C and then incubated with fluoresceine isothiocyanate (FITC)-conjugated anti-rat secondary antibodies (eBioscience, San Diego, CA). The nucleus was stained with 4′,6-diamidino-2-phenylindole (DAPI). Images were recorded using a laser scanning confocal microscope (TCS SP5, Leica, Germany).

### Statistical Analysis

Statistical Product and Service Solutions (SPSS) 22.0 was used for the statistical analysis. The counting data are expressed as the mean ± standard deviation. The data sets were compared using analysis of variance. The significance level was set at *P* < 0.05.

## Results

### Morphology and Size Distribution of Nanobubbles

**Figure 2:**
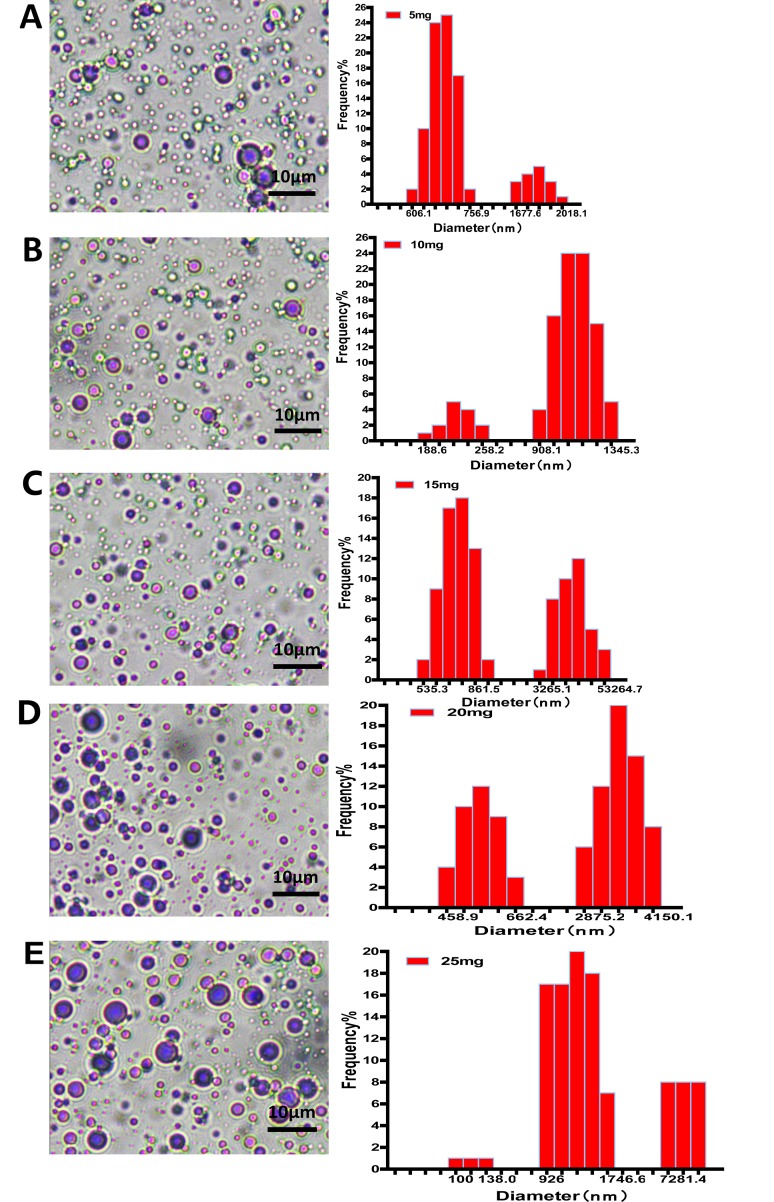
The morphology and particle size distribution of nanobubbles prepared by controlling phospholipid film thickness at 5 mg **(A)**, 10 mg **(B)**, 15 mg **(C)**, 20 mg **(D)**, and 25 mg **(E)**.

The morphology of nanobubbles was observed under oil lens at ×1,000 magnifications. Nanobubbles produced by phospholipid film thicknesses controlling were presented with a dot or sphere with a bright center. DLS analysis shows that these nanobubbles were polydisperse, appearing in two different peaks on the size distribution curves: a higher peak and a lower peak. The higher peaks of nanobubbles fabricated by different film thicknesses appeared at 675.3 nm, 1,124.5 nm, 804.1 nm, 3,425.1 nm, and 976.4 nm, respectively. Meanwhile, the lower peaks appeared at 1,725.2 nm, 204.6 nm, 5,341.2 nm, 528.6 nm, and 7,281.3 nm, respectively (**Figure 2**).

**Figure 3:**
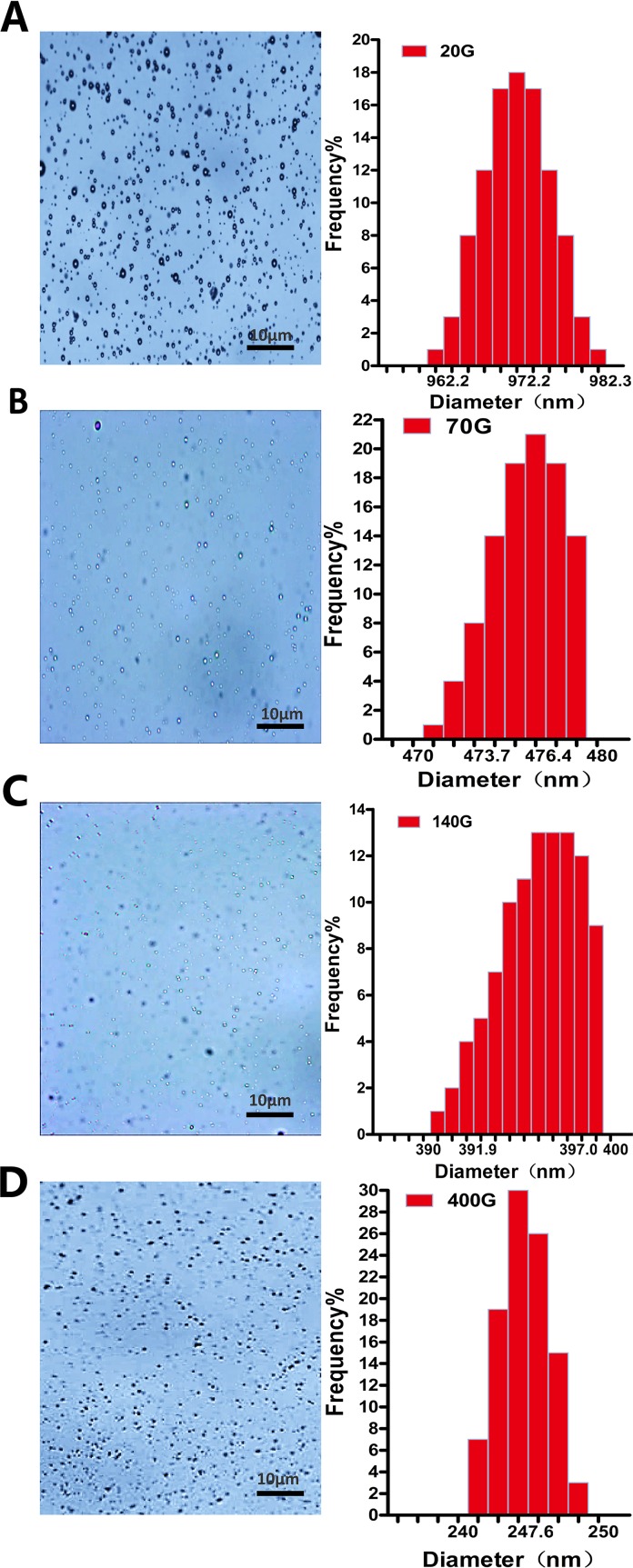
The morphology and particle size distribution of nanobubbles prepared by different centrifugations at 20 g **(A)**, 70 g **(B)**, 140 g **(C)**, and 400 g **(D)**.

Almost all nanobubbles produced by centrifugation were presented with dots. Particle sizing analysis shows that these nanobubbles were monodisperse with only one peak. When the centrifugal conditions were 20 g, 70 g, 140 g and 140 g, the peaks appeared at 972.2 nm, 476.4 nm, 397.0 nm, and 247.6 nm, respectively (**Figure 3**).

### The Mean Diameters and Polydispersity Index of Nanobubbles

**Figure 4:**
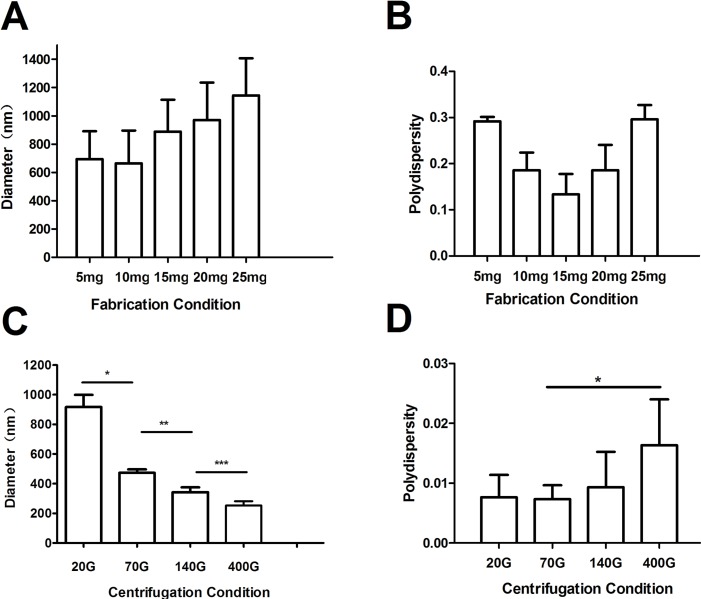
The mean diameter and polydispersity of nanobubbles prepared by controlling phospholipid film thickness and different centrifugations. **(A)** Histogram of the average diameter of the bubbles produced with 5 mg, 10 mg, 15 mg, 20 mg, and 25 mg phospholipid. **(B)** Polydispersity of nanobubble produced with 5 mg, 10 mg, 15 mg, 20 mg, and 25 mg phospholipid. **(C)** Histogram of the average diameter of the bubbles prepared using centrifugation speeds of 20 g, 70 g, 140 g, and 400 g. **(D)** Polydispersity of nanobubbles prepared using centrifugation speeds of 20 g, 70 g, 140 g, and 400 g.

The mean diameters of nanobubbles were approximately increased with the increase of phospholipid film thicknesses. When the thickness was increased from 5 mg to 25 mg, the mean diameters were 723.9 ± 125.7 nm, 734.8 ± 117.6 nm, 977.2 ± 165.9 nm, 1027.5 ± 227.3 nm, and 1141.4 ± 131.8 nm, respectively (**Figure 4A**). However, the mean diameters of nanobubbles had a tendency to decrease as the centrifugal speed increased. When the centrifugal speed was increased from 20 g to 400 g, the mean diameters were 971.3 ± 11.5 nm, 475.2 ± 5.7 nm, 395.8 ± 5.5 nm, and 246.1 ± 8.7 nm, respectively (**Figure 4C**).

PDI is a specific index of particle size distribution. The PDI of nanobubbles produced by various phospholipid film thicknesses were 0.311, 0.211, 0.145, 0.193, and 0.284, respectively (**Figure 4B**). However, the PDIs of the nanobubbles that underwent the centrifugation process were 0.007, 0.005, 0.009, and 0.016, respectively **(Figure 4D**). The PDIs of nanobubbles fabricated by phospholipid film thicknesses controlling were all higher than that by centrifugation (*P* < 0.01), which indicated that the centrifugal process could improve the uniformity of nanobubbles.

### Influence of Centrifugation on Concentration of Nanobubbles

**Figure 5:**
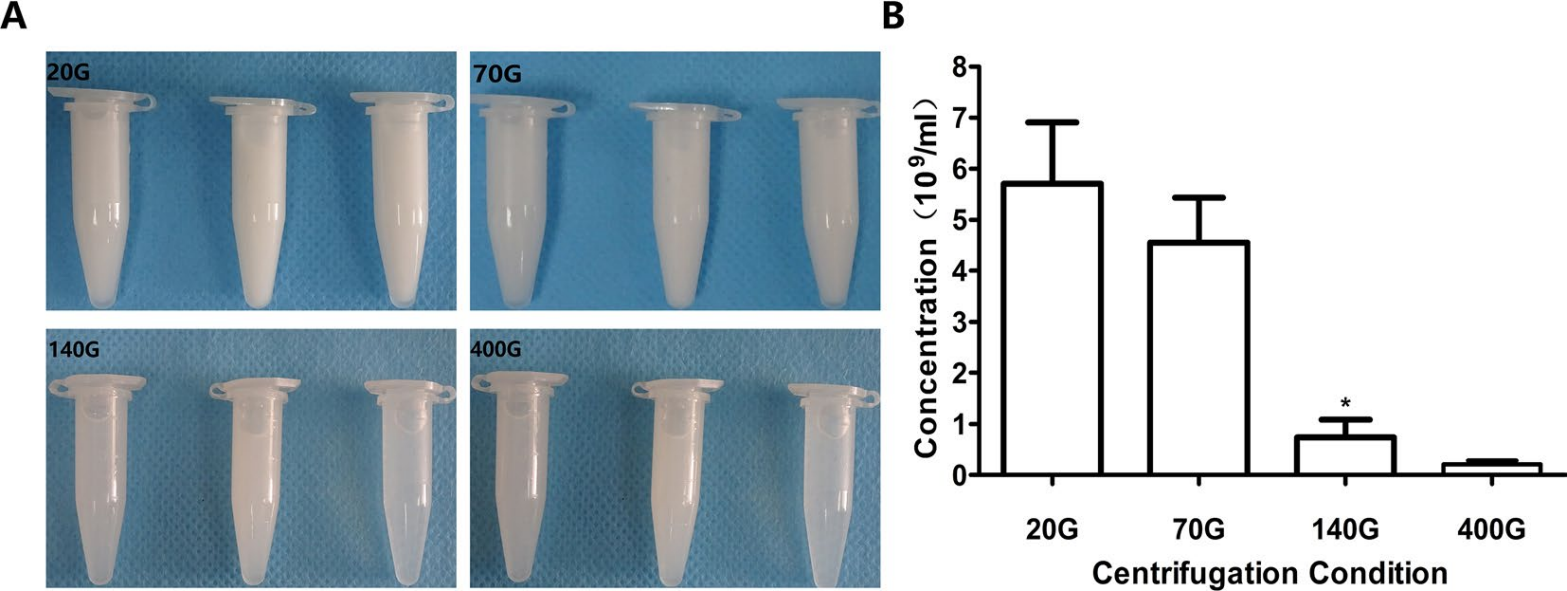
Concentration of nanobubbles at different centrifugation speeds of 20 g, 70 g, 140 g, and 400 g. **(A)** Photos of nanobubbles produced by different centrifugal speed. **(B)** Quantitative analysis of centrifugation of different nanobubbles. *P < 0.05 compared with 20 g.

When the centrifugal speeds were 20 g, 70 g, 140 g, and 400 g, the average concentration of nanobubbles were 6.4 × 10^9^/mL, 5.1 × 10^9^/mL, 1.1 × 10^9^/mL, and 0.32 × 10^9^/mL, respectively. Compared to 20 g, the concentration of nanobubbles at 70 g did not change obviously (*P* > 0.05). However, when the speed reached 140 g, the concentration began to decrease significantly (*P* < 0.01) (**Figure 5**).

As was clear from the above descriptions, 70 g might be the optimal centrifugal condition for nanobubble fabrication, under which the uniform size distribution, appropriate diameter, and relative high concentration of nanobubbles can meet the requirements of most experiments. Therefore, we used nanobubbles fabricated by 70 g in the following experiments.

### Morphology of Nanobubbles Produced by the Optimal Concentration

**Figure 6:**
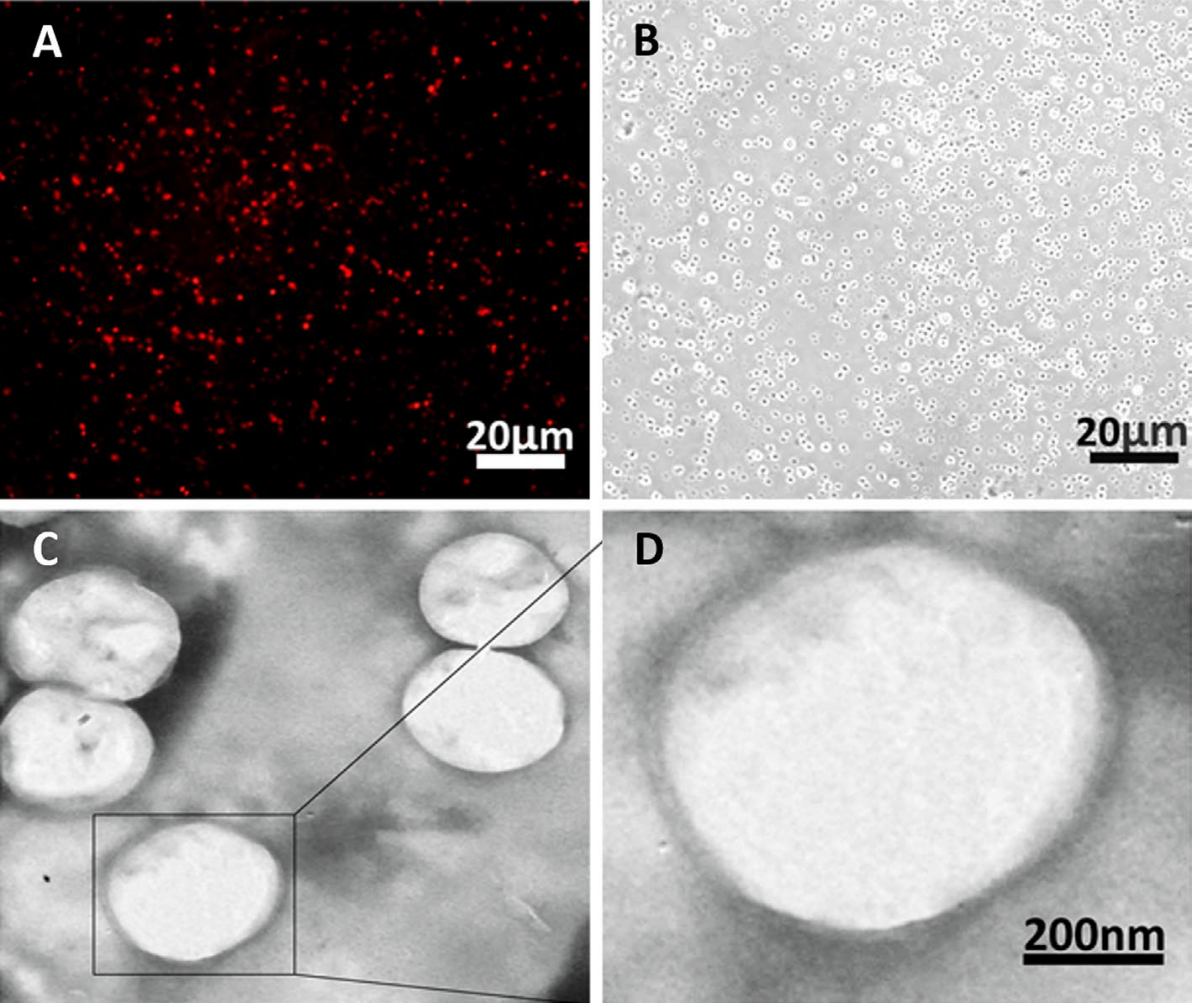
Morphology of nanobubbles produced by optimal centrifugation. **(A)** Fluorescence field of nanobubbles at ×400 magnifications. **(B)** Corresponding bright field of nanobubbles was observed. **(C and D)** The micrograph of nanobubbles obtained by TEM.

The morphology of the nanobubbles prepared by the optimal centrifugal speed was observed under fluorescence microscope at ×400 magnifications and transmission electron microscope (TEM) at ×5,000 magnifications. The DiI-labeled nanobubbles were presented as uniform red dots under a fluorescence microscope and as bright dots in the corresponding bright field (**Figure 6A and B**). Under TEM, the phospholipid (negative control) appeared as a solid sphere (**Figure 6C and D**).

### Stability of Nanobubbles

**Figure 7:**
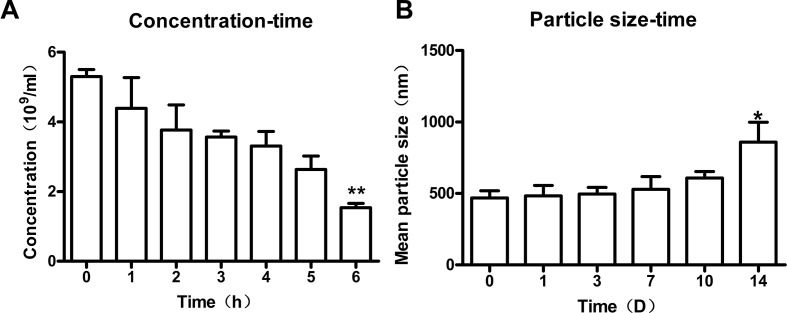
Stability of nanobubbles produced by optimal concentration. **(A)** The concentration of nanobubbles after storage for 1 h, 2 h, 3 h, 4 h, 5 h, and 6h. **(B)** The particle size of nanobubbles after storage for 1 day, 3 days, 7 days, 10 days, and 14 days. 0 h, initial concentration; 0 D, initial size; D, day. **P < 0.01 compared to 0 h; *P < 0.05 compared to 0 D.

To study the stability of nanobubbles, the changes in concentration and size of nanobubbles were monitored at 25°C. The overall trend of nanobubble concentration was downward with the increasing storage time. The initial concentration (0 h) was about 5.4 ± 1.2 × 10^9^/mL. After storage for 1, 2, 3, 4, 5, and 6 h, the concentration changed to 4.3 ± 0.9× 10^9^/mL, 3.7 ± 0.7 × 10^9^/mL, 3.5 ± 0.2 × 10^9^/mL, 3.3 ± 0.4 × 10^9^/mL, 2.6 ± 0.4 × 10^9^/mL, and 1.5 ± 0.3 × 10^9^/mL, respectively (**Figure 7A**). However, only the concentration at 6 h was statistically different compared with that at 0 h (P < 0.01), which indicated that the concentration of nanobubbles could remain unchanged for up to 6 h.

Moreover, the size of nanobubbles tended to be on the rise generally with the increasing storage time. The initial mean diameter of nanobubbles (0 day) was about 475.2 ± 5.7 nm. After storage for 1 day, 3 days, 7 days, 10 days, and 14 days, the diameters changed to 483.3 ± 29.0 nm, 495 ± 19.1 nm, 528.6 ± 35.8 nm, 544.9 ± 37.6 nm, and 859.3 ± 55.9 nm, respectively (ဂ**Figure 7B**). However, there was a significant change in nanobubble diameter until 14 days (*P* < 0.05), which indicated that the diameter of nanobubbles could be kept unchanged for up to 14 days.

### *In Vitro* and *In Vivo* Contrast Enhancement Abilities of Nanobubbles

**Figure 8:**
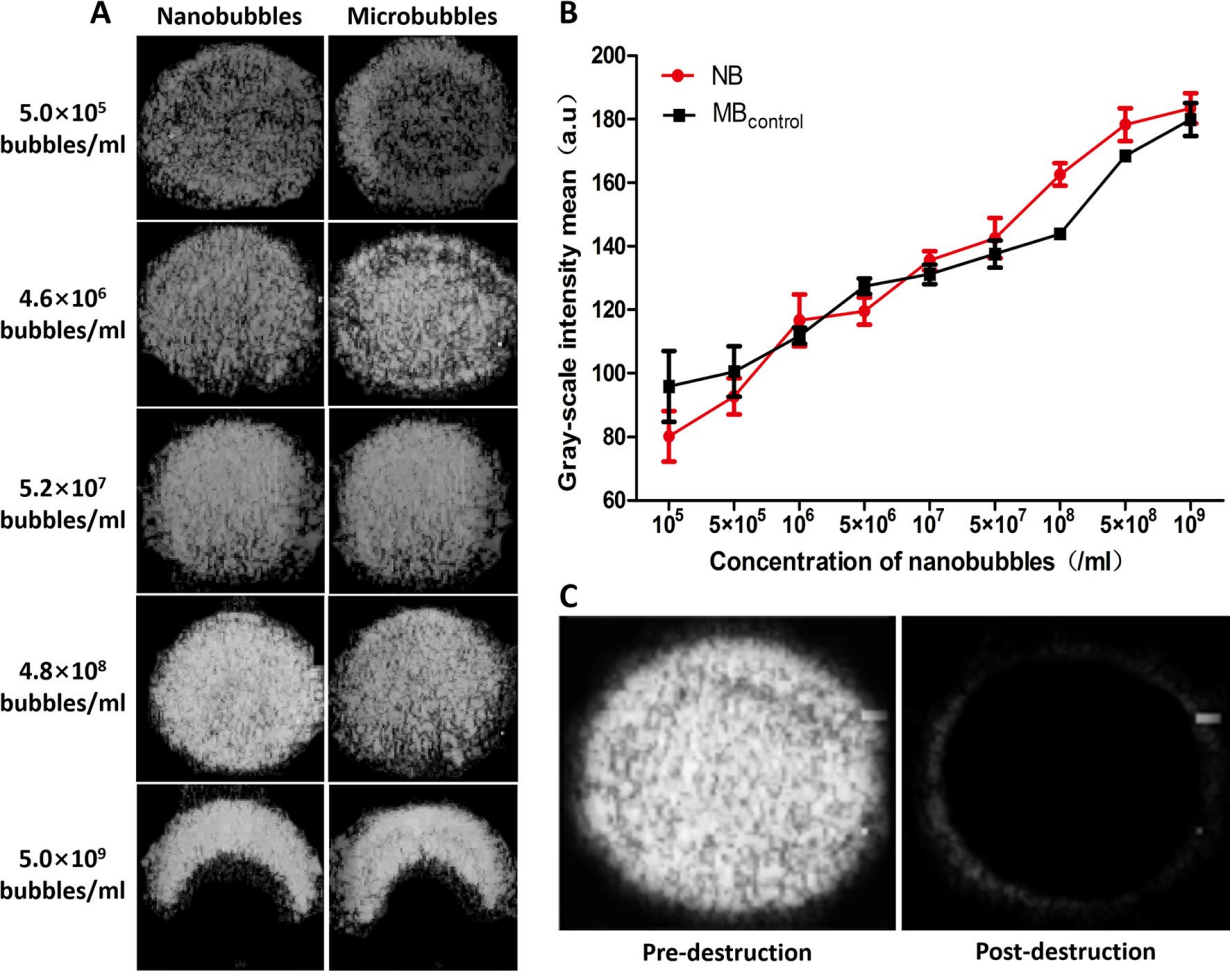
*In vitro* ultrasound image enhancement. **(A)** Representative ultrasound images of nanobubbles and microbubbles *in vitro*. **(B)** Comparison of contrast intensity between nanobubbles and microbubbles at different concentrations. **(C)** Pre- and postdestruction of NBs at low-frequency ultrasound exposure.

*In vitro*, ultrasound images were acquired at various bubble concentrations using diagnostic high-frequency ultrasound (7 MHz). The contrast intensity of nanobubbles became stronger with the increase of nanobubble concentration. When the concentration reached 10^9^/mL, because of the posterior attenuation generated by the strong reflex of the anterior nanobubbles close to the transducer, the ultrasonogram of contrast imaging was presented as a crescent. The performance of nanobubbles was similar to that of microbubbles *in vitro* (**Figure 8A**). Quantitative analysis shows that no significant difference was observed between the signal enhancements of the nanobubbles and microbubbles at each concentration (*P* > 0.05) (**Figure 8B**). After high-power ultrasound exposure, the grayscale intensity of nanobubbles decreased sharply because the nanobubbles were destroyed (**Figure 8C**).

**Figure 9:**
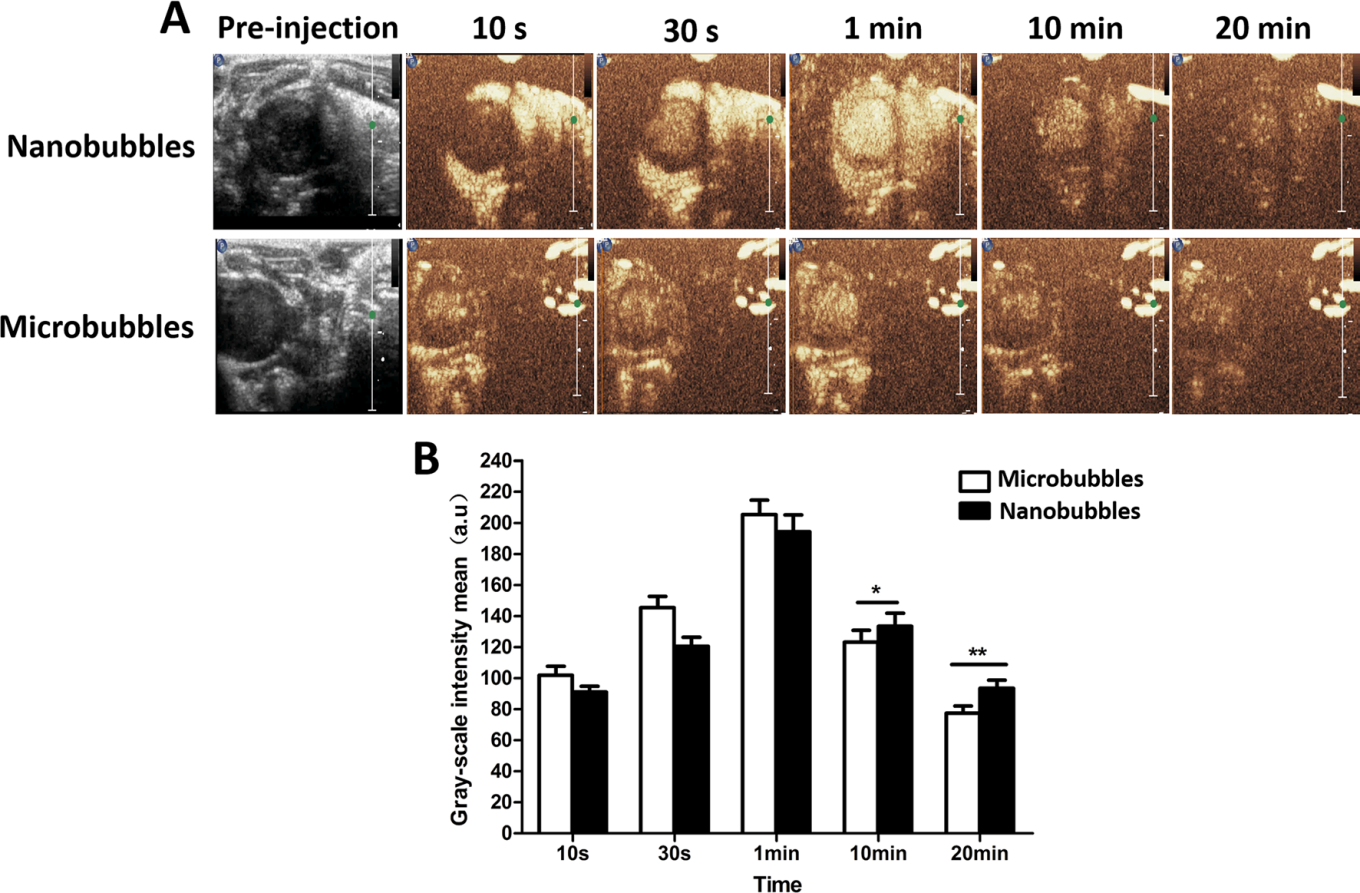
The left ventricular opacification (LVO) in rats. **(A)** The representative LVO images of nanobubbles and microbubbles. **(B)** Grayscale intensity comparison between nanobubbles and microbubbles at the same time point.

In the LVO in rats, the contrast intensity of nanobubbles gradually became stronger with time at the beginning. It reached the peak at about 1 min after intravenous injection and subsequently decreased. The *in vivo* imaging performance of nanobubbles was similar to that of microbubbles (**Figure 9**).

### Passive Targeting Ultrasound Imaging in Tumors

**Figure 10:**
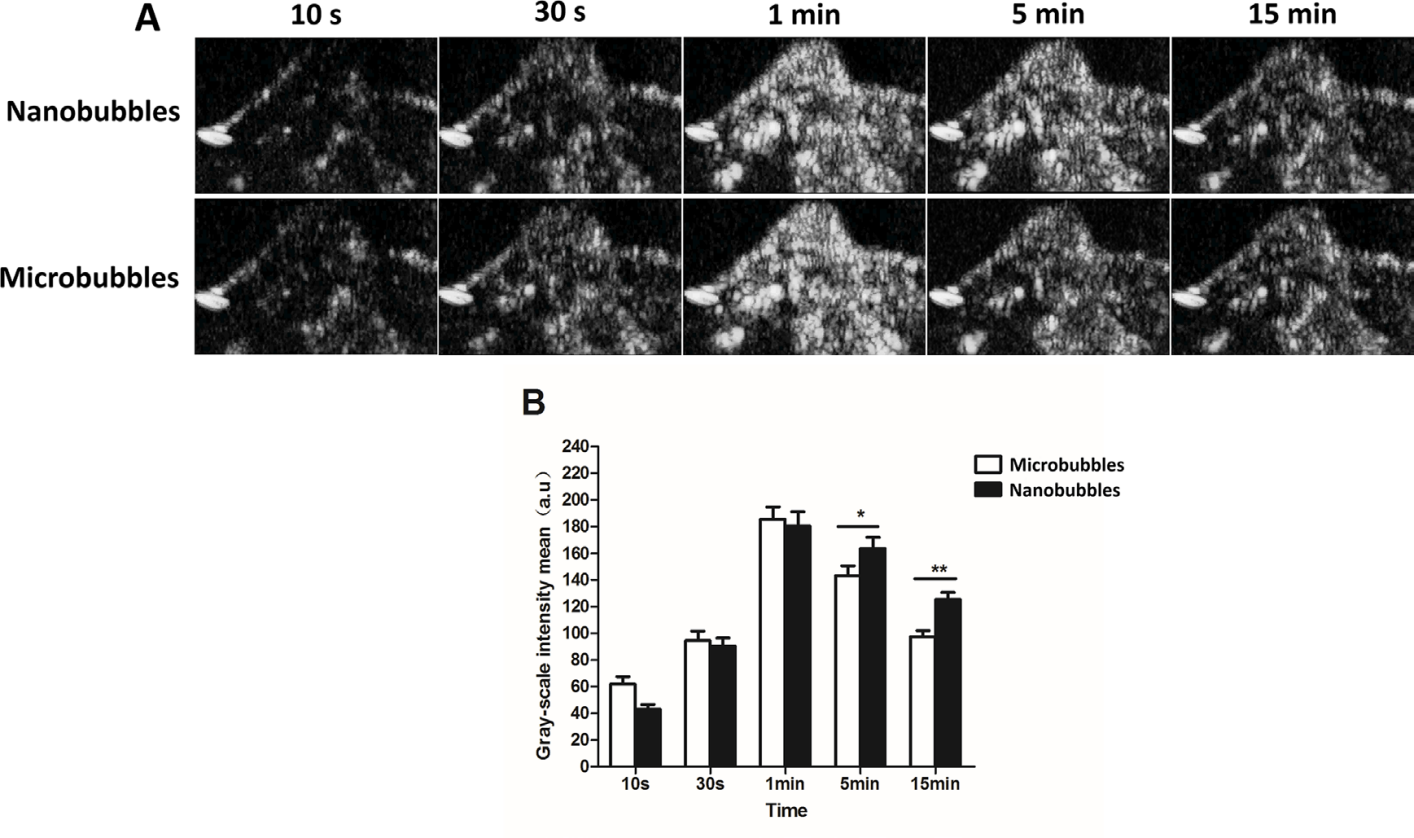
Passive targeting ultrasound imaging of nanobubbles in tumors. **(A)** Representative ultrasound images of nanobubbles and microbubbles. **(B)** The quantitative comparison of imaging capability between nanobubbles and microbubbles at different time points.

Contrast imaging was carried out on 10 tumor-carrying BALB/c mice. No animals died during the experiment. After injecting nanobubbles *via* the tail vein, the signal of nanobubbles became stronger with time and reached the peak at about 1 min, and then declined gradually (**Figure 10A**). At 10 s, 30 s, and 1 min after injection, the signal intensity of nanobubbles was comparable to that of microbubbles (*P* > 0.05). However, at 5 min and 15 min, the grayscale intensity of nanobubbles was significantly higher than that of microbubbles (5 min: 163.5 ± 8.3 a.u. vs. 143.2 ± 7.5 a.u., *P* < 0.01; 15 min: 125.4 ± 5.2 a.u. vs. 97.3 ± 4.6 a.u., *P* < 0.01) (**Figure 10B**). It demonstrated that the duration of contrast enhancement of nanobubbles was significantly longer than that of microbubbles.

### Location of Nanobubbles in Tumors

**Figure 11:**
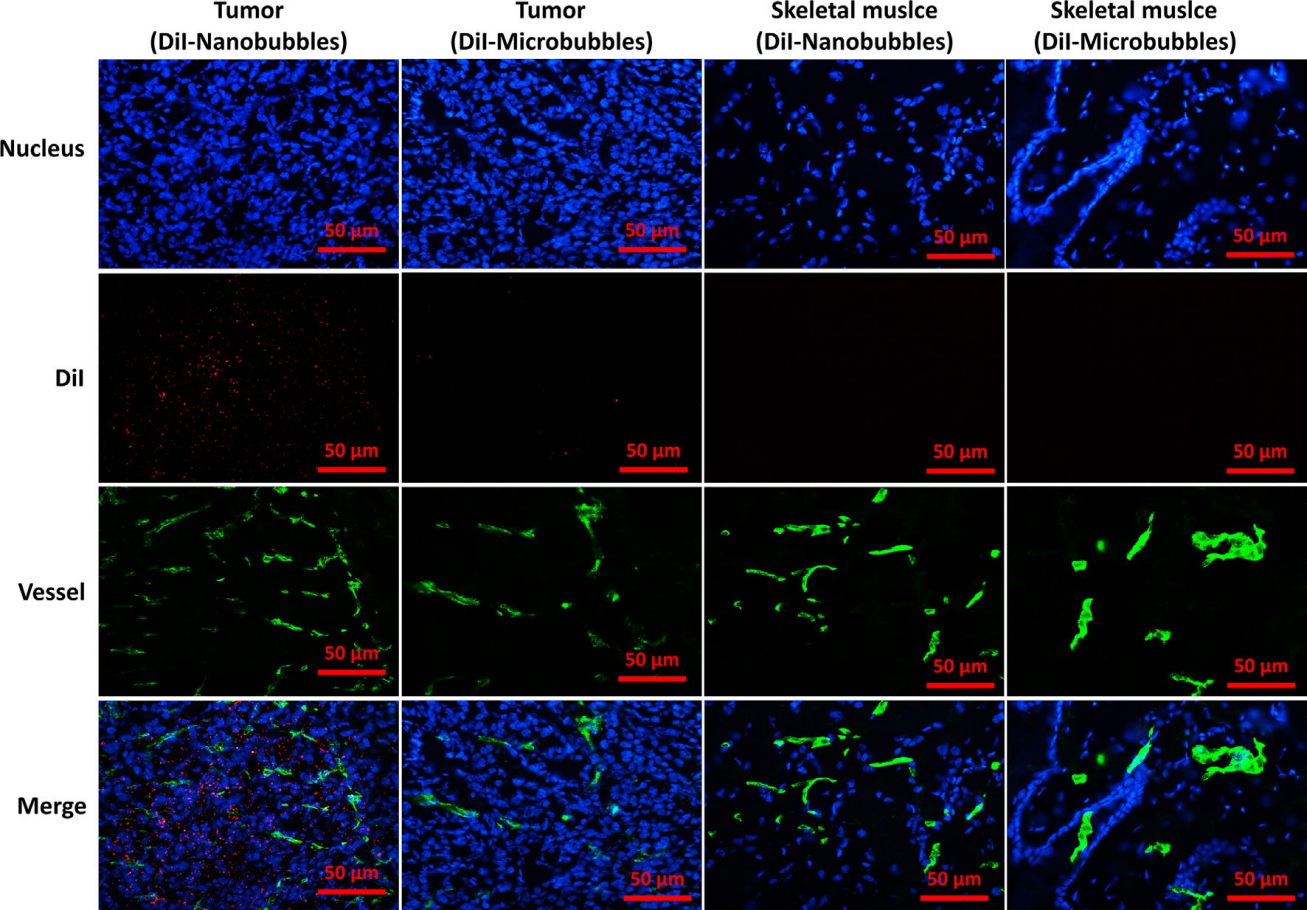
Confocal laser-scanning microscopy images of frozen sections after vessels and nuclear labeling. A considerable number of DiI-labeled nanobubbles were observed in the intercellular space, whereas DiI-labeled microbubbles were hardly visible in tumors. Both DiI-labeled nanobubbles and microbubbles were difficult to detect in skeletal muscle.

The location of DiI-labeled nanobubbles or microbubbles in tumors was observed by CLSM, and skeletal muscle was used as control. Several DiI-labeled nanobubbles (red) were present in the extravascular and intercellular space of the tumor tissues. However, DiI-labeled microbubbles were hardly detected in tumors. In the skeletal muscle sections, however, DiI-labeled nanobubbles and microbubbles were both rare (**Figure 11**).

### Biocompatibility Tests and Cytotoxicity Assay

**Figure 12:**
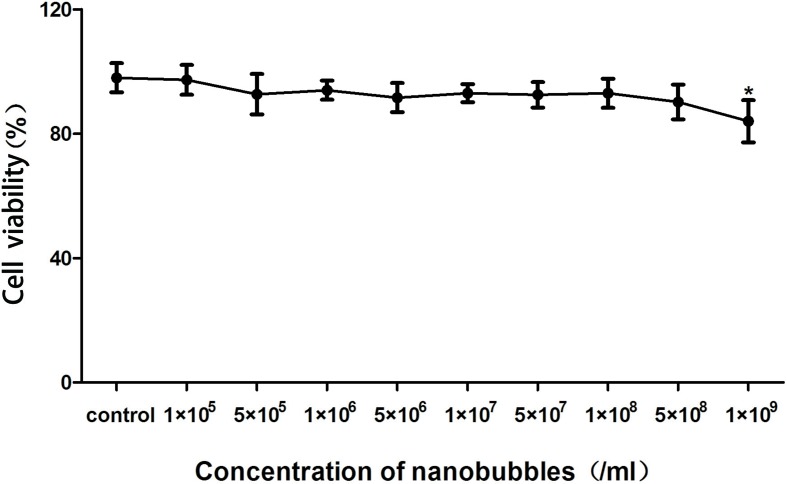
*In vitro* biocompatibility evaluation of nanobubbles using Cell Counting Kit-8 (CCK-8) assay. Control, cells incubated with phosphate buffer saline (PBS) at the same volume.

**Figure 13:**
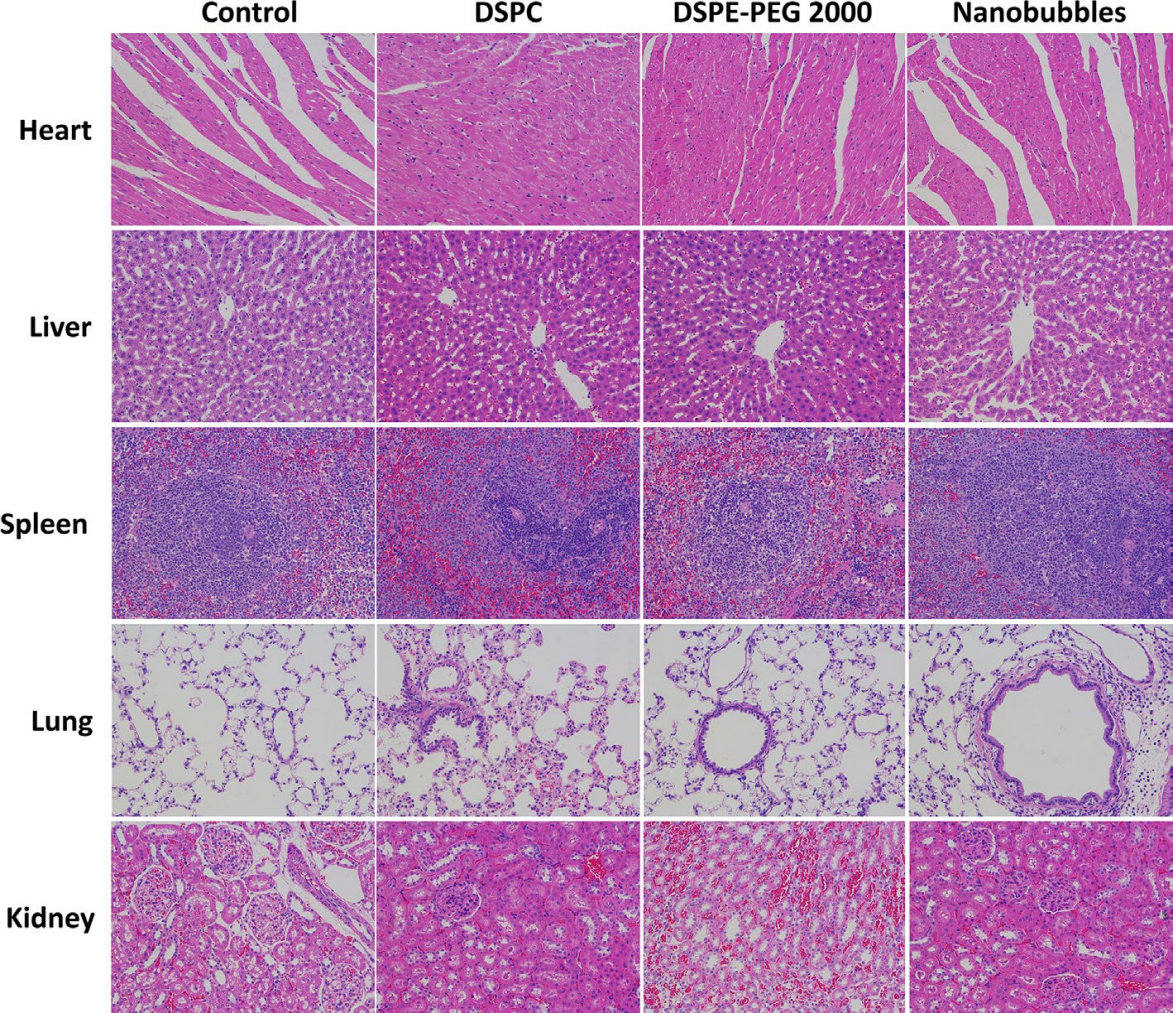
*In vivo* toxicity evaluation of nanobubbles by HE staining of heart, liver, spleen, lung, and kidney (×200 magnification). Control, rats were treated by PBS injection at the same volume.

After incubation with 10^5^–10^9^/mL (nine groups) nanobubbles for 8 h, the viability of bEnd.3 cells was calculated by CCK-8. When the concentration of nanobubbles ranged from 10^5^ to 10^8^/mL, the cell viability had no significant differences compared with the control group (*P* > 0.05). When the concentration went up to 10^9^/mL, the cell viability declined (*P* < 0.05), but remained greater than 85% (**Figure 12**). The *in vivo* cytotoxicity assay showed that after injection of DSPC, DSPE-PEG 2000, or nanobubbles, no structural abnormality was observed in the major organs (heart, liver, spleen, lung, and kidney) in HE-stained slices (**Figure 13**).

## Discussion

Gas-filled bubbles are commonly used as echo-enhancers in ultrasonic diagnosis and as drug-loading vehicles in therapy (McEwan et al., 2015; Huang et al., 2017; Snipstad et al., 2017). Compared to microbubbles that were trapped in the blood pool, nanoscale bubbles (nanobubbles) are promising contrast agents for extravascular ultrasonic imaging and drug delivery (Guvener et al., 2017; Liu et al., 2017). However, research on the preparation condition of nanobubbles with uniform distribution is still in the initial stages (Perera et al., 2017). Thus, we investigated the influence of centrifugation on the character of nanobubbles and compared it with controlling phospholipid film thickness in this study.

The thickness of phospholipid was regarded as a critical factor for nanobubble diameters (Cai et al., 2015). We indeed found that the nanobubble diameter was obviously influenced by phospholipid film thickness. And the diameters seemingly increased with the rise of phospholipid film thickness. However, the uniformity of particle size was relatively poor. DLS analysis shows that the PDI values were high, and there were two peaks on the size distribution curve of different phospholipid film thicknesses. One peak was located at the nanoscale field, and one was located at the microscale field, which means that the bubble suspension acquired by controlling phospholipid film thickness was actually the mixture of nanobubbles and microbubbles. It was also confirmed using a microscope. Under the ×1,000 oil lens, some bubbles presented punctiform and some appeared as a sphere with a bright center. Moreover, the mean diameters were all higher than 700 nm at 5–25 mg of phospholipid, which means that the chance of passing through the tumor pore was poor. Therefore, an additional purification process was needed to separate nanobubbles from the mixture bubble suspension.

Centrifugation was an ideal method of purification. During centrifugation, larger-sized bubbles were separated from mixtures faster. Thus, in theory, if the conditions are set properly, we could obtain desired nanobubbles at any size we wanted. In our study, we found that the nanobubble diameters seemingly decreased with the rise of centrifugal speed. When the speed reaches 400 g, nanobubbles with a mean diameter at 246.1 ± 8.7 nm were obtained. Size uniformity was significantly improved after centrifugation. Compared to controlling phospholipid film thickness, the PDIs of nanobubbles after centrifugation were obviously smaller. The size distribution of nanobubbles were unimodal under different centrifugal conditions. However, the nanobubble concentration was negatively correlated with centrifugal speed. When the centrifugal speed was greater than 70 g, the concentration declined sharply. At a centrifugation of 70 g, the optimal nanobubbles were acquired, with an excellent PDI of 0.005, a mean diameter of 475.2 ± 5.7 nm, and a concentration of 5.4 ± 1.2 × 10^9^/mL. Taking into account the bubble size, PDI, and concentration of all centrifugation conditions, we considered that nanobubbles acquired by 70 g would be most suitable for tumor imaging and drug delivery.

Next, we further studied the characteristics of nanobubbles produced by 70 g centrifugal speed. Fluorescence imaging and TEM further confirmed their morphology and particle size. The size of nanobubbles could remain unchanged for 10 days. However, the concentration was markedly decreased after 6 h. Even so, this stability could still meet the requirements of most experiments. Under *in vitro* and *in vivo* ultrasound imaging, nanobubbles have shown optimal contrast enhancement abilities, which were similar to those of microbubbles. After high-power ultrasound exposure, the attenuation of grayscale intensity indicated that nanobubbles were destroyed, which illustrated that nanobubbles have a good acoustic response and could be used for drug delivery, just like microbubbles did.

Then, the passive targeting ability of nanobubbles was verified by ultrasound contrast imaging in tumor tissues. The contrast duration of the nanobubbles was significantly longer than that of microbubbles. CLSM imaging revealed that DiI-labeled nanobubbles penetrated through endothelial gaps and accumulated in the tumor, and red fluorescence was observed outside vessels. However, limited by their particle size, few microbubbles were present in tumor tissues. These phenomena could explain the ultrasound imaging performance. Because the endothelial gaps of normal tissue are less than 7 nm, neither nanobubbles nor microbubbles were observed in skeletal muscles. As a result, there was passive targeting of the nanobubbles to tumors.

Finally, the biosecurity of nanobubble was confirmed by the CCK-8 assay and histopathology. Phospholipids that made up nanobubbles are known to be low-toxicity materials. C_3_F_8_ is also nontoxic, which can be expelled through the lungs freely. Therefore, the nanobubbles were safe to use in cell studies and *in vivo* ultrasound imaging.

Nanobubbles have been widely used in ultrasound molecular imaging and drug/gene targeting delivery. When conjugated with specific ligands, nanobubbles can be used as a probe in ultrasound molecular imaging of various diseases, such as tumor (Lv et al., 2018), allograft rejection (Liu et al., 2018), and so on. Compared with microbubbles, nanobubbles can migrate from vasculature to the extravascular target site, which greatly expanded the application range of ultrasound molecular imaging. Similar to microbubbles, nanobubbles can also load drugs or genes for therapy. Because of their smaller size, nanobubbles can evade clearance by the reticuloendothelial system to a certain extent and have a longer retention time than microbubbles. As a result, nanobubbles can promote more drug/gene aggregation, especially in tumors because of the EPR effect (Wu et al., 2018). Therefore, this study provided a reference method for the preparation of stabilized nanobubbles with uniform particle size.

## Conclusion

Nanobubbles show promise in tumor imaging and therapy. However, preparing uniform nanobubbles with a desirable size distribution remains a challenge. Lipid nanobubbles prepared by the thin-film hydration method are commonly used currently. But there is still confusion about centrifugation and controlling phospholipid film thickness. In this study, we proved that, for the particle size and homogeneity of nanobubbles, centrifugation was better than controlling phospholipid film thickness. In addition, 70 g may be a relatively suitable centrifugal speed for pure nanobubbles preparation, which exhibited uniform size distribution, excellent passive targeting ability in tumors, and potential for therapy. In addition, it should be noted that centrifugation may generate a certain amount of material waste. However, we believe that this waste can be minimized by optimizing the formulation of lipid materials and centrifugation conditions, which is one of our further research projects in the future.

## Ethics Statement

This study was carried out in accordance with the recommendations of the Animal Care and Use Committee of Huazhong University of Science and Technology. The protocol was approved by the Animal Care and Use Committee of Huazhong University of Science and Technology.

## Author Contributions

JZ and YC completed the main experiment and wrote the first draft of the paper together. CD prepared Figures 2 and 3. LZ and ZS prepared Figures 4 and 5. JW analyzed the data. YY, WH, and QL copyedited the manuscript. MX designed the research. All authors have reviewed the final version of the manuscript and approved it for publication.

## Funding

This work was supported by the National Natural Science Foundation of China (grant nos. 81530056, 81727805, 81801715, 81501494, 81771851, and 81701716) and the HUST Interdisciplinary Innovation Team (0118530300).

## Conflict of Interest Statement

The authors declare that the research was conducted in the absence of any commercial or financial relationships that could be construed as a potential conflict of interest.

The handling editor and reviewer XW declared their involvement as co-editors in the Research Topic, and confirm the absence of any other collaboration.
